# Preoperative Thyroid Peroxidase Antibody Predicts Recurrence in Papillary Thyroid Carcinoma: A Consecutive Study With 5,770 Cases

**DOI:** 10.3389/fonc.2022.881024

**Published:** 2022-05-09

**Authors:** Weibin Wang, Liping Wen, Shitu Chen, Xingyun Su, Zhuochao Mao, Yongfeng Ding, Zhendong Chen, Yiran Chen, Jiaying Ruan, Jun Yang, Jie Zhou, Xiaodong Teng, Thomas J. Fahey, Zhongqi Li, Lisong Teng

**Affiliations:** ^1^Department of Surgical Oncology, The First Affiliated Hospital, Zhejiang University School of Medicine, Hangzhou, China; ^2^Department of General Surgery, The Children’s Hospital, Zhejiang University School of Medicine, Hangzhou, China; ^3^Department of Medical Oncology, The First Affiliated Hospital, Zhejiang University School of Medicine, Hangzhou, China; ^4^Department of Nuclear Medicine, The First Affiliated Hospital, Zhejiang University School of Medicine, Hangzhou, China; ^5^Department of Pathology, The First Affiliated Hospital, Zhejiang University School of Medicine, Hangzhou, China; ^6^Department of Surgery, New York Presbyterian Hospital and Weill Medical College of Cornell University, New York, NY, United States

**Keywords:** thyroid peroxidase antibody, papillary thyroid carcinoma, recurrence, chronic lymphocytic thyroiditis, autoimmune thyroiditis

## Abstract

**Background:**

Thyroid autoimmunity is common in papillary thyroid carcinoma (PTC) and was believed to confer a better prognosis; however, controversy still remains. This study aimed to investigate the prognostic value of chronic lymphocytic thyroiditis (CLT) and preoperative thyroid peroxidase antibody (TPOAb) in PTC patients.

**Methods:**

A retrospective analysis was performed on 5,770 PTC patients who underwent surgical treatment with pathologically confirmed PTC in our institution between 2012 to 2016. The patients were divided into groups with respect to the coexistence of CLT or preoperative TPOAb levels. The clinicopathological characteristics and disease-free survival (DFS) rates were compared between the groups.

**Results:**

The coexistence of CLT was likely to have bilateral, multifocal tumors. Particularly, PTC patients with TPOAb++ (>1,000 IU/L) had a larger tumor size (*p* = 0.007) and higher rates of bilaterality and multifocality than those with TPOAb− (TPOAb< 100 IU/L), while for lymph node metastasis and extrathyroidal extension, there is no statistical difference. Tumor recurrence was found in 15 of 425 (3.5%), 9 of 436 (2.1%), and 56 of 3,519 (1.6%) patients with TPOAb++, TPOAb+, and TPOAb−, respectively (*p* = 0.017). On univariate analysis, TPOAb++ was correlated with tumor recurrence, with a hazard ratio of 2.20 [95% confidence interval (CI), 1.25–3.89], which remained as an independent risk factor at 1.98 (95% CI, 1.10–3.55) on multivariate analysis. PTC patients with TPOAb++ had the lowest DFS rates (96.5 *vs.* 97.9 *vs.* 98.4%, *p* = 0.020).

**Conclusion:**

CLT is not a protective factor in PTC patients. We provide initial evidence that the preoperative TPOAb instead predicts recurrence in papillary thyroid carcinoma.

## Introduction

Thyroid cancer ranks as the most common endocrine malignancy, and its overall incidence increases annually (1, 2). Papillary thyroid carcinoma (PTC) is the predominant histological type, representing more than 90% of all thyroid cancer cases (3). Chronic lymphocytic thyroiditis (CLT) is an autoimmune disease accompanied by the presence of thyroid autoantibody in the blood plasma (4). The prevalence of CLT in PTC has been reported to range from 5 to 38% (5–7). However, the relationship between CLT and PTC remains in dispute. Early studies suggested that preexisting CLT promoted the tumorigenesis of PTC (8–10), but it appeared to confer a better prognosis (8, 11–15). In contrast, some other studies showed that CLT did not interfere with prognosis or even predicted poorer outcomes (16–18).

Thyroid peroxidase antibody (TPOAb) is a serological marker of CLT (19). Its levels can be measured preoperatively and quantitatively and may represent the degree of thyroid inflammation (19, 20). An elevated TPOAb level has been reported to be potentially associated with the development of thyroid cancer (21). However, whether preoperative TPOAb levels correlate with recurrence in PTC patients remains unclear.

In the current study, we aimed to investigate the clinical value of coexistent CLT in a cohort of 5,770 consecutive PTC patients. We also evaluated the clinicopathologic and prognostic significance of preoperative TPOAb levels in these cases. The large sample size provided us with an opportunity to study the prognostic effect of TPOAb levels in PTCs.

## Materials and Methods

### Subjects

**Figure 1:**
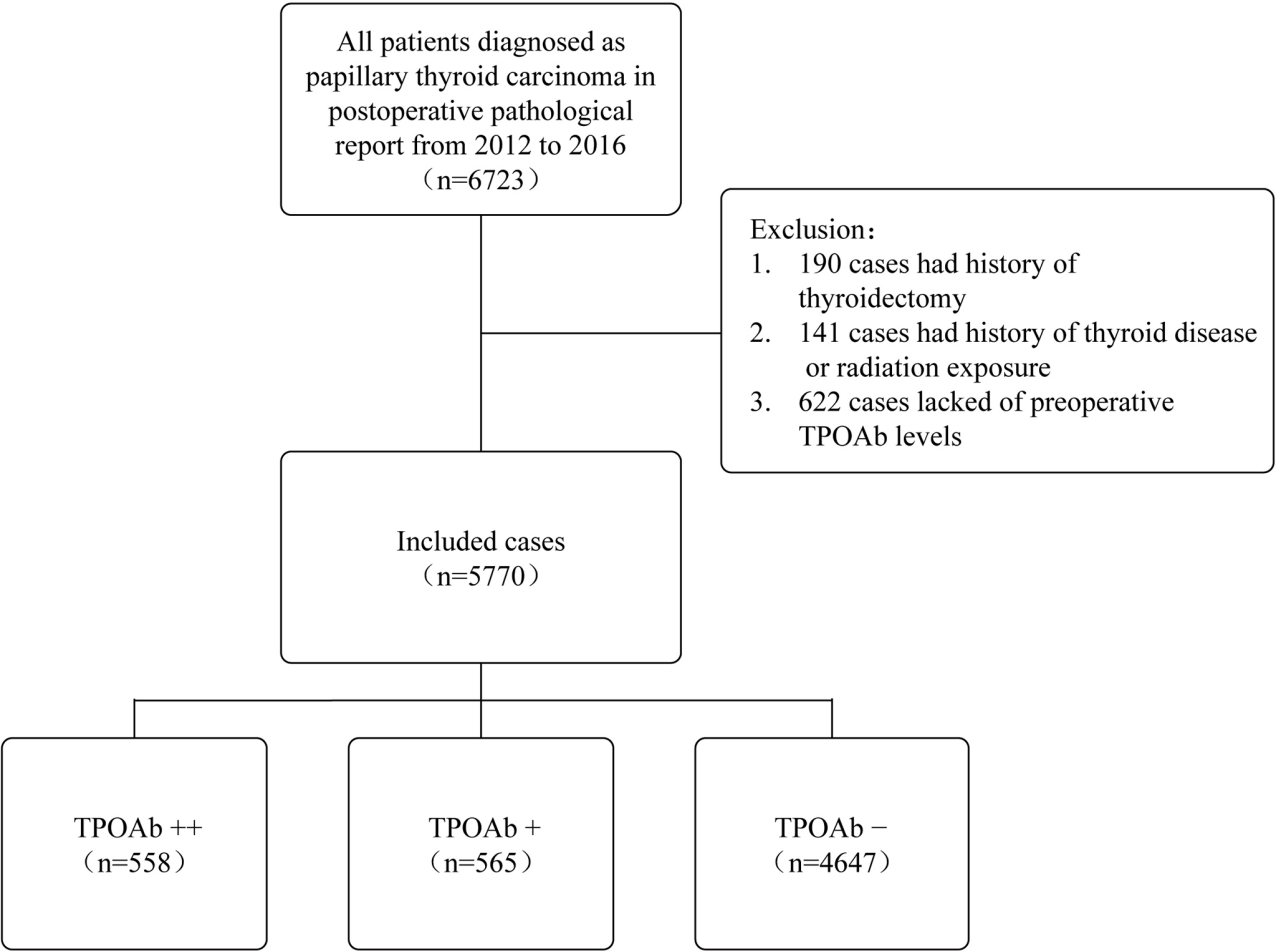
Case selection process for this study. TPOAb, thyroid peroxidase antibody; TPOAb−, 0 < TPOAb ≤ 100 IU/L; TPOAb+, 100 < TPOAb ≤ 1,000 IU/L; TPOAb++, TPOAb > 1,000 IU/L.

The medical records of 6,723 patients who underwent total or hemithyroidectomy with a final histopathological diagnosis of PTC at the First Affiliated Hospital, Zhejiang University School of Medicine (Hangzhou, China), from 2012 to 2016, were retrospectively reviewed. In total, 190 patients with a history of thyroidectomy, 141 patients with a history of thyroid disease or radiation exposure, and 622 patients lacking preoperative TPOAb levels were excluded. A total of 5,770 patients were finally included in the retrospective study (**Figure 1**). Among them, 2,775 (48.1%) cases underwent hemi-thyroidectomy, and 2,995 (51.9%) cases underwent total thyroidectomy. This study was approved by the Institutional Review Board of the First Affiliated Hospital, Zhejiang University School of Medicine.

### Data Collection

The following clinical variables were collected from the registry: age, gender, preoperative serum levels of free triiodothyronine (FT3), free thyroxine (FT4), thyrotrophin (TSH), and TPOAb. TPOAb was measured by an automated chemiluminescent immunoassay system (Advia Centaur; Siemens, Munich, Germany) at 1 to 2 days before surgery. The postoperative pathological reports, including tumor size, bilaterality, multifocality, extrathyroidal extension (ETE), lymph node metastasis (LNM), and tumor stage (AJCC 8th), were also studied. According to previous studies, CLT was diagnosed based on either preoperative TPOAb higher than 100 IU/L or the presence of diffuse lymphocytic infiltration in the surrounding thyroid tissue (12, 22). For multifocal tumors, the maximum diameter was recognized as the tumor size. Additionally, we defined TPOAb+ when its serum level fell in between 100 and 1,000 IU/L (100 < TPOAb ≤ 1,000 IU/L) and defined TPOAb++ when the serum level was 10 times higher than the normal value (TPOAb > 1,000 IU/L). The normal ranges for the serum levels of FT4, FT3, TSH, and TPOAb were 10.45–24.38 pmol/L, 2.77–6.31 pmol/L, 0.380–4.340 mIU/L, and 0–100.0 IU/L, respectively, at our institution.

At the end of the study, 4,380 patients were available for survival analysis. These patients were followed postoperatively with measurements of serum thyroglobulin and thyroglobulin antibody, neck ultrasound, and iodine-131 whole-body scans to monitor for disease recurrence and survival. The mean follow-up was 3.5 years (range, 1–5 years). A total of 696 (15.9%) cases received adjuvant radioactive iodine (RAI) treatment. Moreover, 80 of 4,380 patients (1.8%) were diagnosed with recurrent disease or metastases, including 17 in the thyroid bed, 60 in the cervical lymph nodes, and 3 with lung metastases. In most patients, recurrence was confirmed by pathologic examination, while 10 patients were diagnosed based on increased thyroglobulin levels and imaging evidence from iodine-131 scans.

### Groups and Comparisons

The clinicopathological features and disease outcomes were assessed between the 2 groups according to the coexistence of CLT or not. Based on the preoperative TPOAb levels, we also divided all PTC patients into those with TPOAb−, TPOAb+, and TPOAb++ to investigate the prognostic values of different preoperative TPOAb levels.

### Statistical Analysis

The SPSS 25.0 software was used for all statistical analysis. Statistical significance was defined as a 2-tailed *p*-value of less than 0.05. Continuous variables were presented as mean ± SD, and categorical variables were presented as the number of cases, with percentage (%). Pearson’s chi-square test was used for categorical variables, and Student’s *t*-test or one-way analysis of variance was used for continuous variables. The variables associated with clinical outcomes were evaluated using Cox proportional hazard models in univariate/multivariate analyses. Disease-free survival (DFS) curves were constructed using the Kaplan–Meier method, and the log-rank test was used to compare DFS.

## Results

### Coexistent CLT Did Not Predict a Better Outcome in PTC Patients

CLT was present in 1,482 of 5,770 (25.7%) PTC patients, and their clinicopathologic characteristics are shown in **Table 1**. The PTC patients with CLT were significantly correlated with a younger age (43.9 ± 11.6 *vs.* 45.6 ± 11.8, *p* < 0.001), female gender (88.2 *vs.* 71.5%, *p* < 0.001), more bilateral (25.1 *vs.* 20.6%, *p* < 0.001) and multifocal (35.2 *vs.* 28.5%, *p* < 0.001) tumors as well as a higher proportion of early stage (AJCC 8th stage I: 93.6 *vs.* 91.3%, *p* = 0.005). No difference was observed between the 2 groups regarding the prevalence of ETE (10.1 *vs.* 10.3%, *p* = 0.819) and the frequency of LNM (39.8 *vs.* 38.1%, *p* = 0.239). For disease recurrence, however, we even found that PTC patients with CLT had a relatively higher recurrence rate than those without CLT, with borderline significance (2.5 *vs.* 1.6%, *p* = 0.059).

**Table 1:** Clinicopathologic features of PTC patients with and without CLT.

Variables	PTC with CLT (*n* = 1,482)	PTC alone (*n* = 4,288)	*p*
Age (years)			
Mean ± SD	43.9 ± 11.6	45.6 ± 11.8	<0.001^a^
Age <55 years	1,217 (82.1%)	3,272 (76.3%)	<0.001^b^
Female, %	1,307 (88.2%)	3,064 (71.5%)	<0.001^b^
Bilaterality, %	372 (25.1%)	882 (20.6%)	<0.001^b^
Multifocality, %	522 (35.2%)	1,222 (28.5%)	<0.001^b^
Tumor sizec, *D* ≥10 mm, %	561 (37.9%)	1,425 (33.2%)	0.001^b^
ETE, %	150 (10.1%)	443 (10.3%)	0.819^b^
LNM, %	590 (39.8%)	1,633 (38.1%)	0.239^b^
AJCC 8th I, %	1,387 (93.6%)	3,913 (91.3%)	0.005^b^
Recurrence, %	28/1,132 (2.5%)	52/3,248 (1.6%)	0.059^b^

Statistically significant differences were defined as p < 0.05.

PTC, papillary thyroid carcinoma; CLT, chronic lymphocytic thyroiditis; D, diameter; ETE, extrathyroidal extension; LNM, lymph node metastasis.

a Student’s t-test.

b Pearson’s chi-square test.

### Preoperative TPOAb Levels Correlate With Aggressive Clinicopathological Features

TPOAb is the best serological marker of CLT. To further investigate the role of CLT in PTC, we stratified the PTC patients into TPOAb++, TPOAb+, and TPOAb− groups according to the preoperative TPOAb levels (**Table 2**). PTCs with higher TPOAb levels tended to exhibit a younger age (42.8 ± 11.7 *vs.* 44.5 ± 11.3 *vs.* 45.5 ± 11.8, *p* < 0.001), female preponderance (86.9% *vs.* 86.7% *vs.* 73.1%, *p* < 0.001), higher rates of bilaterality (28.7% *vs.* 24.4% *vs.* 20.6%, *p* < 0.001) and multifocality (38.2% *vs.* 35.4% *vs.* 28.6%, *p* < 0.001). However, there was no difference among the 3 groups regarding the prevalence of ETE (8.8% *vs.* 11.5% *vs.* 10.3%, *p* = 0.320) or LNM (40.1% *vs.* 39.3% *vs.* 38.2%, *p* = 0.632). Furthermore, we found that TPOAb ++ group was significantly characterized by younger age, female preponderance and more aggressive features: higher rates of bilaterality, multifocality, tumor size ≥ 10mm and recurrence. Additionally, we compared the levels of TPOAb with the pathological features on inflammation degree. Oxyphilic metaplasia, follicular atrophy or follicular disruption, which indicate high degree of thyroid inflammation (19), were more frequently found in TPOAb++ group. Accompanied higher TSH levels indicated more severe destruction of thyroid follicular cells in these patients. (**Supplementary Table S1**).

**Table 2:** Clinicopathologic features of patients with PTC stratified by the status of TPOAb.

Variables	TPOAb			*P*	*p* for TPOAb–^a^ *vs.*	
	++ (*n* = 558)	+ (*n* = 565)	– (*n* = 4647)		TPOAb+^a^	TPOAb++^a^
Age (years)						
Mean ± SD	42.8 ± 11.7	44.5 ± 11.3	45.5 ± 11.8	<0.001^b^	0.066	<0.001
Age <55 years	474 (84.9%)	463 (81.9%)	3,552 (76.4%)	<0.001^c^	0.003	<0.001
Female, %	485 (86.9%)	490 (86.7%)	3,396 (73.1%)	<0.001^c^	<0.001	<0.001
Bilaterality, %	160 (28.7%)	138 (24.4%)	956 (20.6%)	<0.001^c^	0.034	<0.001
Multifocality, %	213 (38.2%)	200 (35.4%)	1,331 (28.6%)	<0.001^c^	0.001	<0.001
Tumor size, *D* ≥10 mm	221 (39.6%)	208 (36.8%)	1,557 (33.5%)	0.007^c^	0.117	0.004
ETE, %	49 (8.8%)	65 (11.5%)	479 (10.3%)	0.320^c^	0.380	0.259
LNM, %	224 (40.1%)	222 (39.3%)	1,777 (38.2%)	0.632^c^	0.627	0.589
AJCC 8th I, %	530 (95.0%)	528 (93.5%)	4,242 (91.3%)	0.004^c^	0.081	0.003
Recurrence, %	15/425 (3.5%)	9/436 (2.1%)	56/3,519 (1.6%)	0.017^c^	0.408	0.005
TSH	2.20 (0.01–62.07)	1.94 (0.01–28.00)	1.66 (0.01–45.16)	<0.001^d^	<0.001	<0.001
FT4	15.0 (5.5–24.6)	15.4 (7.31–29.1)	15.4 (4.4–50.6)	<0.001^d^	0.353	0.043
FT3	4.7 (1.9–9.8)	4.7 (3.2–9.0)	4.8 (2.8–20.1)	<0.001^d^	<0.001	0.750

Statistically significant differences were defined as p < 0.05.

PTC, papillary thyroid carcinoma; TPOAb, thyroid peroxidase antibody; D, diameter; ETE, extrathyroidal extension; LNM, lymph node metastasis.

a TPOAb–, 0 < TPOAb ≤ 100 IU/L; TPOAb+, 100 < TPOAb ≤ 1,000 IU/L; TPOAb++, TPOAb > 1,000 IU/L.

b One-way analysis of variance.

c Pearson’s chi-square test.

d Mann–Whitney U-test.

### Preoperative TPOAb ++ Was an Independent Risk Factor for Disease Recurrence

We then investigated the prognostic value of preoperative TPOAb levels. Tumor recurrence was found in 15 of 425 (3.5%), 9 of 436 (2.1%), and 56 of 3,519 (1.6%) PTC patients in the TPOAb++, TPOAb+, and TPOAb– groups, respectively (*p* = 0.017, **Table 2**). Univariate analysis revealed that age <55, bilaterality, multifocality, tumor size ≥10 mm, ETE, LNM, total thyroidectomy, RAI, and TPOAb++ were significantly associated with tumor recurrence (**Table 3**). By multivariate Cox analysis, LNM [hazard ratio (HR), 3.90; 95% CI, 1.20–8.00; *p* < 0.001], RAI (HR, 4.06; 95% CI, 2.20–7.49; *p* < 0.001), and TPOAb++ (HR, 1.98; 95% CI, 1.10–3.55; *p* = 0.023) were identified as independent risk factors for tumor recurrence.

**Table 3:** Association between recurrence and TPOAb levels of PTC patients.

Variables	Univariate analysis		Multivariate analysis	
	HR (95% CI)	*P*	HR (95% CI)	*p*
Age <55	2.43 (1.17–5.04)	0.017	1.69 (0.80–3.56)	0.165
Female	0.77 (0.48–1.24)	0.278	0.96 (0.59–1.57)	0.871
Bilaterality	2.31 (1.48–3.61)	<0.001	0.77 (0.38–1.59)	0.484
Multifocality	2.46 (1.59–3.82)	<0.001	1.66 (0.82–3.37)	0.161
Tumor size, *D* ≥10 mm	3.70 (2.33–5.88)	<0.001	1.44 (0.85–2.43)	0.172
ETE	3.24 (1.97–5.34)	<0.001	1.44 (0.84–2.47)	0.191
LNM	9.82 (5.20–18.56)	<0.001	3.90 (1.20–8.00)	<0.001
Total thyroidectomy	3.49 (2.016–6.032)	<0.001	1.08 (0.54–2.16)	0.820
RAI	10.32 (6.49–16.41)	<0.001	4.06 (2.20–7.49)	<0.001
TPOAb+^a^	1.29 (0.64–2.61)	0.479	1.19 (0.58–2.44)	0.634
TPOAb++^a^	2.20 (1.25–3.89)	0.007	1.98 (1.10–3.55)	0.023

Statistically significant differences were defined as p < 0.05.

TPOAb, thyroid peroxidase antibody; PTC, papillary thyroid carcinoma; D, diameter; ETE, extrathyroidal extension; LNM, lymph node metastasis; RAI, radioactive iodine treatment.

a TPOAb– is the reference for TPOAb+ and TPOAb++; TPOAb–, 0 < TPOAb ≤ 100 IU/L; TPOAb+: 100 < TPOAb ≤ 1,000 IU/L; TPOAb++: TPOAb > 1,000 IU/L.

**Figure 2:**
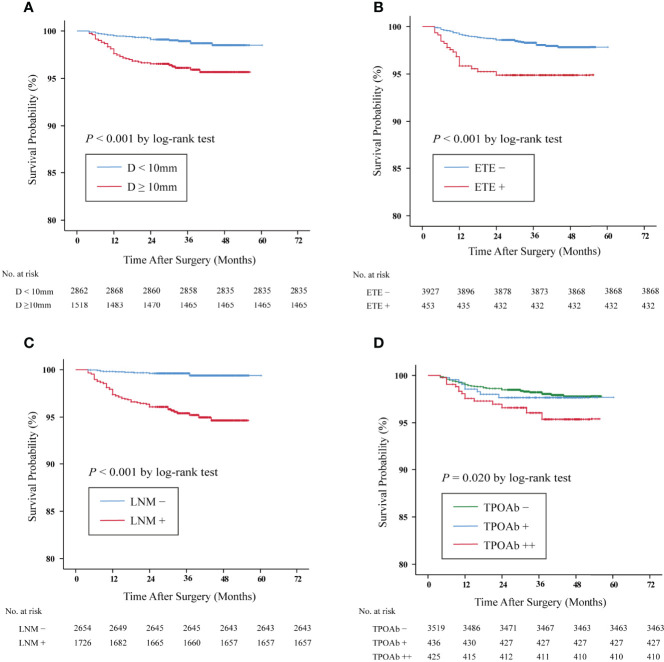
Kaplan–Meier survival curves of disease-free survival (DFS) in papillary thyroid carcinoma (PTC) patients. The DFS rates of PTC patients with **(A)** tumor size ≥10 mm *vs.* <10 mm: 96.5 *vs.* 99.1%, *p* < 0.001. **(B)** Extrathyroidal extension (ETE) + *vs.* ETE–, 95.4 *vs.* 98.5%, *p* < 0.001. **(C)** Lymph node metastasis (LNM)+ *vs.* LNM–: 96.0 *vs.* 99.6%, *p* < 0.001. **(D)** The DFS rates of the TPOAb++ group were significantly lower than those of the TPOAb– group (*p* = 0.020 by log-rank test, TPOAb++ *vs.* TPOAb −, *p* = 0.005; TPOAb++ *vs.* TPOAb+, *p* = 0.195; TPOAb+ *vs.* TPOAb−, *p* = 0.476). TPOAb−, 0 < TPOAb ≤ 100 IU/L; TPOAb+, 100 < TPOAb ≤ 1,000 IU/L; TPOAb++, TPOAb > 1,000 IU/L.

Furthermore, we compared the Kaplan–Meier DFS curves with respect to the risk factors identified above. The 3.5-year DFS rates of those patients with tumor size ≥10 mm (96.5 *vs.* 99.1%, *p* < 0.001, shown in **Figure 2A**), ETE (95.4 *vs.* 98.5%, *p* < 0.001, shown in **Figure 2B**), and LNM (96.0 *vs.* 99.6%, *p* < 0.001, shown in **Figure 2C**) were significantly lower than those of the control group. It is noteworthy that the lowest DFS curve was documented in the TPOAb++ group compared with the TPOAb+ and TPOAb– groups (96.5 *vs.* 97.9% *vs.* 98.4%, *p* = 0.020, shown in **Figure 2D**).

## Discussion

CLT is frequently found in PTC, but its prognostic implication in PTC remains an active focus of research and is still under debate. Previous studies displayed that coexistent CLT was associated with a lower rate of ETE and lymph node metastasis in PTC patients (12, 23), while very recently, Lee et al. found more frequent multifocality and ETE in PTC patients coexisting with CLT, but with a similar recurrence rate than those without CLT (17). In the present study with 5,770 cases, we demonstrated that CLT was not a protective factor for PTC patients. Coexistent CLT was instead attributed to a larger tumor size, bilaterality, multifocality, and a relatively lower DFS rate. Notably, a high preoperative TPOAb level (>1,000 IU/L) was identified as an independent risk factor for tumor recurrence in PTC patients. Our findings may be partially explained by a recent study which implied that lymphovascular and perineural invasion was more common in the PTCs with CLT group (24).

TPOAb is measurable preoperatively and regarded as a sensitive marker of CLT or thyroid autoimmunity (25). The discrepancy concerning the role of coexistent CLT in predicting the prognosis of PTC could partially result from the different degree of thyroid inflammation. Previous studies showed that an elevated TPOAb level might contribute to the development of thyroid cancer (26, 27), while a recent meta-analysis found that although positive TPOAb was associated with an increased risk of thyroid cancer, this association did not exist in some subgroups (28). They argued that the relation between positive TPOAb and the risk of thyroid cancer remained to be elucidated (28). On the other hand, elevated TPOAb appeared as a protective factor for lymph node metastasis (21, 29). However, Shen et al. argued that TPOAb positivity was a risk indicator for more metastatic cervical lymph nodes (30). Recently, Song et al. demonstrated that positive TPOAb was associated with less tumor recurrence in PTC patients (31). Not conducting a subgroup analysis according to the TPOAb values might account in part for these inconsistent conclusions. The levels of TPOAb have been demonstrated to correlate with the degree of thyroid inflammation in autoimmune thyroiditis (19, 20). A higher level of TPOAb may represent a severe degree of inflammation. Here we graded the severity of autoimmune inflammation by preoperative TPOAb levels and found that TPOAb++ was an independent risk factor for disease recurrence. PTC patients with TPOAb++ exhibited the shortest DFS, indicating that a higher degree of thyroid inflammation could predict tumor recurrence in PTC patients.

The extent of initial thyroidectomy is a major concern when treating PTC patients, and it is usually determined by multiple factors. Here we found a much higher incidence of bilaterality and multifocality in patients with CLT (25.1 *vs.* 20.6 and 35.2 *vs.* 28.5%, respectively). Particularly, in patients with TPOAb++, the possibility of bilaterality and multifocality was as high as 28.7 and 38.2%. Therefore, total thyroidectomy may be favored in these PTC patients. However, it seems not rational to perform a more aggressive prophylactic cervical lymph node dissection for PTCs with CLT since the rate for lymph node metastasis was almost the same as that of patients without CLT (39.8 *vs.*38.1%).

The current study has some certain limitations. Primarily, it is a retrospective, single-institution study. In addition, because of the favorable prognosis of PTC, our follow-up period is not long enough to uncover the true prognostic significance. We plan to continue this study to obtain longer outcomes. Another limitation is that we do not have thyroglobulin antibody data, which could serve as another clinical marker for CLT (32). Lastly, the current study lacks information of genetic alterations like BRAF and RET/PTC rearrangements, which correlate with CLT and may impact the patients’ outcomes.

In conclusion, our large retrospective cohort study demonstrated that CLT is not a protective factor in PTC patients. We instead provide new evidence that a high TPOAb level (>1,000 IU/L) correlates aggressive features, including larger tumor size, bilaterality, and multifocality, and is an independent risk factor for tumor recurrence. Hence, a preoperative evaluation of the TPOAb level is worthwhile for risk stratification and post-treatment surveillance in PTC patients.

## Data Availability Statement

The raw data supporting the conclusions of this article will be made available by the authors without undue reservation.

## Ethics Statement

The studies involving human participants were reviewed and approved by The Clinical Research Ethics Committee of the First Affiliated Hospital, Zhejiang University School of Medicine. Written informed consent to participate in this study was provided by the participants’ legal guardian/next of kin.

## Author Contributions

WW, LW, SC, LT, and ZL designed the current study and wrote the manuscript. WW, LW, ZM, and YD conducted the statistical analyses. WW, LW, SC, XS, ZM, YD, ZC, YC, JR, JY, JZ, and XT created the original databases to collect the clinicopathological data. TF, ZL, and LT reviewed and revised the manuscript. All authors contributed to the article and approved the submitted version.

## Funding

This research was funded by the National Natural Science Foundation of China (numbers 81772853, 81972495, 81902719 and 82102758), the National Natural Science Foundation of Zhejiang (number LY18H160012), and the Key Project of Scientific and Technological Innovation of Zhejiang Province (number 2015C03031).

## Conflict of Interest

The authors declare that the research was conducted in the absence of any commercial or financial relationships that could be construed as a potential conflict of interest.

## Publisher’s Note

All claims expressed in this article are solely those of the authors and do not necessarily represent those of their affiliated organizations, or those of the publisher, the editors and the reviewers. Any product that may be evaluated in this article, or claim that may be made by its manufacturer, is not guaranteed or endorsed by the publisher.
