# Supplementary material for: Preoperative Thyroid Peroxidase Antibody Predicts Recurrence in Papillary Thyroid Carcinoma: A Consecutive Study With 5,770 Cases
Source: Front Oncol. 2022 May 9;12:881024. doi: 10.3389/fonc.2022.881024 (PMC9124958; doi:10.3389/fonc.2022.881024)
Supplement: Supplementary file 1 [file Table_1.doc]

**Table S1** Association between TPOAb levels and the degree of thyroiditis

| Variables | TPOAb | | | *p* | *p* for TPOAb ++ ^a^ *vs.* | |
| --- | --- | --- | --- | --- | --- | --- |
|  | ++ (n = 350) | + (n = 248) | – (n = 359) |  | TPOAb – ^a^ | TPOAb + ^a^ |
| High-degree CLT ^b^ | 245 (70.0%) | 155 (62.5%) | 148 (41.2%) | **<0.001^c^** | **<0.001** | 0.055 |
| TSH | 2.54 (0.01-62.07) | 2.16 (0.01-28.00) | 1.91 (0.01-9.62) | **<0.001^d^** | **<0.001** | 0.060 |
| FT4 | 15.0 (5.5-24.6) | 15.1 (10.5-26.3) | 15.2 (8.8-29.3) | 0.106 ^d^ | **0.043** | 0.678 |
| FT3 | 4.7 (1.9-6.5) | 4.7 (3.4-8.6) | 4.7 (2.8-9.5) | 0.632 ^d^ | 0.750 | 0.482 |

^a^ TPOAb –: 0 < TPOAb ≤ 100 IU/L; TPOAb +: 100 < TPOAb ≤ 1000 IU/L; TPOAb ++: TPOAb > 1000 IU/L

^b^ High-degree CLT was based on the coexisting oxyphilic metaplasia, follicular atrophy or follicular disruption

^c^ Pearson’s Chi square test

^d^ Mann-Whitney U test
